# On Obstetric Practice

**Published:** 1815-10

**Authors:** R. Kinglake

**Affiliations:** of Taunton.


*For the London Medical and Physical Journal.*

### *On Obstetric Practice;* by Dr. R. Kinglake, of Taunton.

THE legitimate objects of the practice of midwifery should be most precisely defined, to obviate the hurtful speculations into which practitioners, anxious to leave nothing undone, are liable to be betrayed. To suppose an inadequacy in nature to accomplish every requisite respecting the birth of the human species, is to imagine that chance, and not consummate wisdom, is concerned in this most important event. The works of nature are universally perfect; and the institutions and provisions of the animal economy are more particularly distinguished by the utmost exactness. Deviations from the general competency of nature in the function of parturition, may, and actually do, in common with all other natural laws, occasionally present; but, when this is the case, the validity of the principle that asserts the fundamental sufficiency of nature to her own deliverance, is in no respect compromised. It will, on these occasions, be found, that the cause of an occurring difficulty has been accidental, and is connected with circumstances that clearly point out both the necessity and mode of relief. Whether the occurring impediment be of a mechanical or physiological character, it should be gravely estimated by the attending circumstances, and its claim for manual aid be decided accordingly. When a hasty decision is made on these occasions, it is not that which the nature of the case authorises, but which an erroneous or precipitate judgment has suggested; gested: hence the importance of considering well what the legitimate objects of the obstetric art really are; that useful assistance, instead of irreparable injury, may be occasionally afforded.

Misapprehension of the circumstances that constitute a real necessity for assisting the natural efforts of parturition, has proved a fertile source of mischievous practice. The difficulty must be real, and not imaginary, that demands assistance; and the consideration of it should not be attended by either impatience, or an undue opinion of the capabilities of art, when resorted to in aid of natural insufficiency. The practical maxim should be, to wait sufficiently long clearly to ascertain that the accustomed competency of nature to the task is thwarted and counteracted by either mechanical or physiological obstacles, imperiously requiring the interposition of art. Either a preconceived opinion, or a persuasion arising from the experience of the case, may suggest a notion of the indispensible necessity of manual assistance. Excepting in cases of wrong presentation, in hæmorrhages, or convulsions, no sufficient warranty is afforded for resorting to instrumental means to accelerate that which is probably speedy enough for the welfare of the patient. It is at once unjustifiable and highly censurable to refer to instrumental help, or rather instrumental violence and injury, when the bodily strength and mechanical provision for delivery are not insuperably opposed to a safe and seasonable issue of the case. Suitable delay, and proper confidence in the resources of nature, are powerful auxiliaries in parturient difficulties. Numerous instances have and are occurring which may be cited in reprobation of the familiarity with which attempts at instrumental delivery are made. I say attempts, for it is credible, in the large majority of these officious and unfeeling trials, that the endeavour does not succeed; but this failure is no advantage to the suffering patient;—her feelings are, indeed, aggravated by disappointment, and the mental and bodily distress occasioned by the fruitless effort, may be easier conceived than described.

The abuse into which the employ of instruments in the obstetric art has fallen, has partly resulted from an inconsiderate desire to be doing something, where, in strictness, there is nothing to be done; and, in a still greater degree, by the misconception which the patient and her gossiping attendants have of what ought to be attempted for her relief. The formality of indiscriminately attending lying-in women, necessarily suggests the notion, with uninformed persons, that direct aid is to be afforded. This supposition induces an impatience that would not be discovered, were it generally rally understood that no actual assistance can be given when all circumstances are natural; and that the deviation from this state occurs much too rarely to justify uniform attendance in expectation of them. Bad cases in midwifery are announced by features not to be mistaken; and even on those occasions, the urgency is not so great but there will be time sufficient to procure such special assistance as may be required. In these circumstances, the neighbouring or family surgeon who may deserve that appellation might as confidently be referred to as in cases of fracture, or any other accident, to afford the necessary relief. If real occasion should exist for it, he will enter on the performance of his duty with as much familiarity and safety as he would execute the most common operation in surgery. It would be an essential part of his medical education, to know all the exigencies of the obstetric art; and his science would be only employed in accomplishing the legitimate objects of relief, and in no case be exercised at a period when nature may be either hurtfully counteracted or anticipated, rather than salutarily hastened and assisted.

Man-midwifery, therefore, should be confined to absolute occasions for operative aid, and then the art would be directly efficient. No indefinite waiting would be necessary: assistance would be promptly rendered, and the result would probably prove remedial. The interest of humanity, the dignity of science, and the cause of truth, concur in demanding an abandonment of ordinary midwifery practice, as not less inconsistent with the unmolested comfort of the patient, than the justice of philosophy, and the frankness of undisguised honesty. The usages of whole nations of people in all climates, and under all the different circumstances that can possibly affect European females, attest the correctness of the opinion, that the practice of midwifery, as now pursued, is gratuitous, meritricious, and hurtful; and that it therefore ought to be abandoned, as a busy, intermeddling, and mischievous craft.

*July* 12, 1815.

